# Identification and Sensory Evaluation of 3‑Sulfanylhexyl Propionate and 3‑Sulfanylhexyl Butyrate in Wine

**DOI:** 10.1021/acs.jafc.5c12053

**Published:** 2026-01-30

**Authors:** Florian Kiene, Niël van Wyk, Claus Patz, Andrii Tarasov, Vicky Bäumer, Christoph Schüßler, Rainer Jung, Christian von Wallbrunn, Isak S. Pretorius, Doris Rauhut

**Affiliations:** † Department of Microbiology and Biochemistry, 367317 Hochschule Geisenheim University, Von-Lade-Straße 1, 65366 Geisenheim, Germany; ‡ ARC Centre of Excellence in Synthetic Biology, Department of Molecular Sciences, 95531 Macquarie University, Sydney, NSW 2113, Australia; § Department of Beverage Research, Hochschule Geisenheim University, Von-Lade-Straße 1, 65366 Geisenheim, Germany; ∥ Department of Enology, Hochschule Geisenheim University, Von-Lade-Straße 1, 65366 Geisenheim, Germany

**Keywords:** aroma, thiols, esters, Sauvignon blanc, Scheurebe

## Abstract

Two new polyfunctional thiols, 3-sulfanylhexyl propionate (3SHP) and 3-sulfanylhexyl butyrate (3SHB), were detected in wines, synthesized as reference standards, and their sensory properties were characterized. Fermentations supplemented with short-chain fatty acids (propionic acid and butyric acid) generated 3SHP and 3SHB, which were confirmed by GC-MS using synthetic standards. Analysis of commercial wines from Scheurebe, Sauvignon blanc, and Verdejo further verified the presence of 3SHP and 3SHB, reporting their first identification in wines to our knowledge. To determine the influence of these esters on wine aroma, their odor threshold values (detection and recognition thresholds) were identified. Here, both compounds were described with grapefruit, passionfruit and tropical fruit descriptors. Although concentrations in many commercial samples were below sensory detection thresholds, synergistic sensory interactions with other polyfunctional thiols to the tropical wine aroma can be assumed.

## Graphical abstract

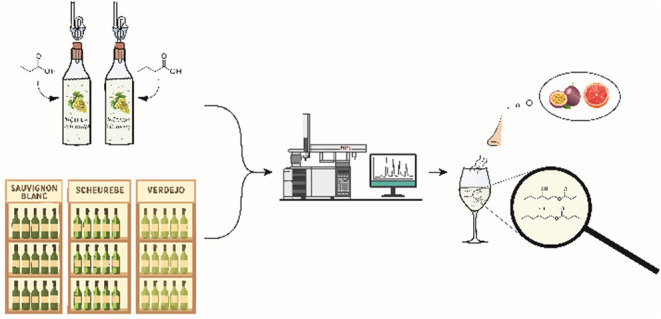

## Introduction

Polyfunctional thiols are volatile sulfur compounds that shape the aroma of wines, particularly in varieties like Sauvignon blanc. The three most commonly researched polyfunctional thiols are 3-sulfanylhexan-1-ol (3SH), its acetate ester 3-sulfanylhexyl acetate (3SHA), and 4-methyl-4-sulfanylpentan-2-one (4MSP). The former two impart grapefruit and passionfruit aromas, whereas the latter imparts box tree and blackcurrant aromas to wine. 3SH and 3SHA are chiral and its enantiomers exhibit different odor qualities.^1^ Due to their low sensory thresholds, detectable at nanogram-per-liter levels, they are character impact compounds in the sensory profile of wine, making them a focal point in wine research.

These compounds originate from nonvolatile precursors in grapes. These precursors include, in particular, l-cysteine and l-glutathione conjugates of 4MSP and 3SH, as well as dipeptide intermediates with l-cysteinylglycine and γ-glutamylcysteine.^2–5^ In addition, 3SH can be released from the aldehyde precursor (GSH-3SH-al) and its bisulfite adduct (GSH-3SH–SO_3_).^6^ Furthermore, 3SH precursors can be formed from (*E*)-2-hexen-1-ol and (*E*)-2-hexenal and 4MSP could arise from mesityl oxide and H_2_S.^7–9^ The concentration of precursors in grape must depends on many factors. Apart from the specific grape variety, various viticultural and enological practices can significantly influence the qualitative and quantitative composition of precursors.^10–12^

During alcoholic fermentation, microbial action through β-lyase activity cleaves these precursors, releasing volatile thiols. The choice of yeast strains, sequential fermentations and higher fermentation temperatures can increase the release of thiols.^13–17^ Recent studies also show the different impacts of cysteinylated and glutathionylated precursors on the release of thiols during fermentation.^18^ However, aging, copper addition, or high O_2_ concentrations in the headspace will decrease the thiol concentration.^10^

The identification and quantification of volatile thiols in foods and beverages are a challenge because of the complex matrices, their extremely low concentrations in the ng·L^–1^ range, and their high reactivity. For these reasons, many different analytical methods have been developed so far.^19^

As already mentioned, the thiol 3SH and its acetate 3SHA are important aroma compounds in wine. However, it can be assumed that 3SH esters are also formed with acids other than acetic acid during fermentation and are present in wine. Besides acetic acid and its esters, wine also contains other microbially derived volatile fatty acids such as propionic acid, butyric acid, isobutyric acid, isovaleric acid, hexanoic acid, octanoic acid, and decanoic acid. Among these, acetic acid is the most abundant, typically occurring at concentrations of 100–500 mg·L^–1^. Propionic acid is present in concentrations of up to 100 mg·L^–1^, and butyric acid is found at levels from 0.4 to 5 mg·L^–1^.^20^

Previously, Waterhouse et al. stated the following: “All carboxylic acids and alcohols in wine can potentially esterify. Since there are dozens (if not hundreds) of both of these compound classes in wines, one could expect to find thousands of different esters in wine, if only at vanishingly small concentrations.”^20^ This statement inspired us to investigate whether unknown esters with low perception thresholds and high influence on wine aroma could be identified.

Since thiols, in particular, can exert a strong influence on wine aroma at very low concentrations, the main objective of this investigation was to use the already well-researched 3SH to determine in preliminary fermentations the formation of corresponding esters through the addition of higher concentrations of fatty acids. Based on the results of previous tests, we investigated the occurrence of other 3SH esters, and identified the two esters 3-sulfanylhexyl propionate (3SHP) and 3-sulfanylhexyl butyrate (3SHB) for the first time in wine and described their sensory impact. To achieve this, fermentation trials with spiked 3SH and fatty acids were carried out, 3SHB and 3SHP were synthesized for analytical confirmation, and a sensory study was conducted to evaluate their sensory impressions. Additional GC-MS measurements of Sauvignon blanc, Scheurebe, and Verdejo wines confirmed the occurrence of 3SHB and 3SHP in real wine samples.

## Materials and Methods

### Chemicals and Biological Reagents

Reagents used, including suppliers and purity, are listed in the Supporting Information.

### Fermentation

For the short fermentation trials, we used a commercially available *Saccharomyces cerevisiae* yeast strain, VIN13 (Anchor Yeast, Cape Town, South Africa). VIN13 was genetically modified (Supporting Information Table S1) to build the strain VIN13­[tnaA_ATF1], which overexpresses its own alcohol acetyltransferase 1 gene (*ATF1*) and expresses a codon-optimized version of the carbon–sulfur lyase from *Escherichia coli* (*tna*A) as previously described.^21,22^ The yeasts were precultured in YPD (10 g·L^–1^ Yeast Extract, 20 g·L^–1^ Peptone, 20 g·L^–1^ glucose) and inoculated in must at a concentration of approximately 1 × 10^6^ cells·mL^–1^ as determined by a hemocytometer. Due to its typically low thiol content, Müller-Thurgau (MT) must was used. The MT must had a total sugar concentration of 193.5 g·L^–1^ (93.4 g·L^–1^ glucose and 100.1 g·L^–1^ fructose). Tartaric acid and malic acid were 5.3 g·L^–1^ and 1.7 g·L^–1^, respectively with a pH of 3.22. The MT grape must’s yeast available nitrogen concentration (YAN) was 113 mg·L^–1^. Fermentations were conducted in 100 mL Schott flasks containing 100 mL of MT must sealed with an airlock containing ∼4 mL of water. In certain cases, 1 μL propionic acid or butyric acid were added to the fermentation at the start along with 1 μL 2-phenylethanol (2PE), pentan-1-ol, hexan-1-ol, or 3-sulfanylhexan-1-ol (3SH). The flasks were incubated at 22 °C for one to 2 days prior to aroma analysis.

### Syntheses of 3-Sulfanylhexyl Propionate (3SHP) and 3-Sulfanylhexyl Butyrate (3SHB)

To synthesize 3SHP and 3SHB, the method for the synthesis of 3-sulfanyl-1-^2^H_2_-hexyl acetate was modified.^23^

An amount of 20 mmol (2.0 equiv) of the corresponding acyl chloride (butyryl chloride or propionyl chloride) was added to a solution of 3-sulfanylhexan-1-ol (1.343 g, 10.0 mmol, 1.0 equiv) in 10 mL dry dichloromethane (DCM) and stirred for 2 h under inert gas atmosphere at room temperature. Then, water (10 mL) followed by 10 mL of a saturated NaHCO_3_ solution were added and extracted with 2 × 10 mL DCM. After drying over Na_2_SO_4_, the solvent was removed by distillation. Purification of the resulting oil was performed by flash chromatography (hexane/DCM, 9:1). The progress of the reaction and the purity of the collected fractions were checked by thin-layer chromatography (silica gel plates, hexane/ethyl acetate (9:1)). The compounds exhibited *R*_f_ values of 0.49 (3SHB) and 0.46 (3SHP), respectively. Spots were visualized by exposure to iodine vapor, and collected fractions were analyzed using GC–MS. This yielded both compounds as intensely fruity-smelling oils (3SHB: 1.53 g, 7.49 mmol, 75%; 3SHP: 1.33 g, 6.94 mmol, 69%) with a purity of >99.9% each (by GC-MS, Supporting Information Figures S1 and S2 for 3SHB and Figures S3 and S4 for 3SHP).

#### 3SHB

MS (EI, 70 eV), *m*/*z* (%): 133 (3), 116 (87), 101 (30), 88 (91), 83 (68), 71 (100), 67 (25), 60 (9), 55 (53), 43 (50), 41 (25) (Supporting Information Figure S5).

^1^H NMR (400 MHz; CDCl_3_) δ = 0.92 (3H, t, *J* = 7.1 Hz, CH_3_), 0.95 (3H, t, *J* = 7.4 Hz, CH_3_), 1.39 (1H, d, *J* = 7.3 Hz, SH), 1.41–1.53 (2H, m, CH_3_ CH_2_CH_2_CH­(SH)), 1.53–1.69 (4H, m, CH_2_), 1.69–2.07 (2H, m, CH_2_), 2.28 (2H, t, *J* = 7.4 Hz, COCH_2_), 2.82–2.93 (1H, m, CH­(SH)), 4.20–4.30 (2H, m, CH­(SH)­CH_2_ CH_2_O) (Supporting Information Figure S7).

^13^C NMR (100 MHz; CDCl_3_) δ = 13.7 (2C, CH_3_), 18.5 (CH_2_), 20.1 (CH_2_), 36.2 (CH_2_), 37.3 (CH­(SH)), 37.8 (CH_2_), 41.2 (CH_2_), 62.0 (CH­(SH)­CH_2_ CH_2_O), 173.6 (CO) (Supporting Information Figures S8 and S9).

RI: 1587 (60 m × 0.25 mmID, 1.0 μm *d*_f_, Rxi-5Sil MS, Restek); 1938 (30 m × 0.25 mmID, 0.25 μm *d*_f_, Stabilwax-DA, Restek GmbH, Bad Homburg, Germany).

#### 3SHP

MS (EI, 70 eV), *m*/*z* (%): 133 (2), 116 (73), 101 (24), 88 (76), 83 (58), 73 (34), 67 (28), 57 (100), 55 (58), 47 (8), 41 (20) (Supporting Information Figure S6).

^1^H NMR (400 MHz; CDCl_3_) δ = 0.92 (3H, t, *J* = 7.1 Hz, CH_3_CH_2_CH_2_CH­(SH)), 1.14 (3H, t, *J* = 7.6 Hz, C­(O)­CH_2_ CH_3_), 1.39 (1H, d, *J* = 7.3 Hz, SH), 1.41 – 1.68 (4H, m, CH_3_ CH_2_ CH_2_CH­(SH)), 1.68–2.07 (2H, m, CH­(SH)CH_2_), 2.33 (2H, q, *J* = 7.6 Hz, C­(O)CH_2_CH_3_), 2.83–2.93 (1H, m, CH­(SH)), 4.20–4.30 (2H, m, CH­(SH)­CH_2_ CH_2_O) (Supporting Information Figure S10).

^13^C NMR (100 MHz; CDCl_3_) δ = 9.1 (CH_3_), 13.7 (CH_3_), 20.1 (CH_2_), 27.6 (CH_2_), 37.3 (CH­(SH)), 37.8 (CH_2_), 41.2 (CH_2_), 62.1 (CH­(SH)­CH_2_ CH_2_O), 174.3 (CO) (Supporting Information Figures S11 and S12).

RI: 1449 (60 m × 0.25 mmID, 1.0 μm d_f_, Rxi-5Sil MS, Restek); 1862 (30 m × 0.25 mmID, 0.25 μm *d*_f_, Stabilwax-DA, Restek GmbH, Bad Homburg, Germany).

### HS-SPME-GC-MS

Detection of esters in the preliminary tests was performed by headspace solid-phase microextraction gas chromatography–mass spectrometry (HS-SPME-GC-MS) measurements according to a method previously published.^24^

### Analysis of Polyfunctional Volatile Thiols

Polyfunctional volatile thiols were quantitated according to a previously published method.^22^

Mass spectral detection was carried out in SIM using EI at 70 eV. Ethyl propiolate (ETP)-derivatized thiols were detected with the following masses and retention times: 4MMB (4-methoxy-2-methyl-2-butanethiol, internal standard)-ETP (*m*/*z* 233, 200, 133; RT = 15.1 min); 4MSP-ETP (*m*/*z* 230, 157, 133; RT = 16.6 min); 3SHA-ETP (*m*/*z* 274, 229, 83; RT = 18.1 min); 3SHP-ETP (*m*/*z* 288, 255, 141, 83; RT = 18.8 min); 3SHB-ETP (*m*/*z* 302, 269, 141, 83; RT = 19.4 min); 3SH-ETP (*m*/*z* 233, 187, 133; RT = 19.8 min).

Calibration was performed in Sauvignon blanc wine (Sb13) with ten concentration levels: (4MSP: 0–156 ng·L^–1^, *R* ^2^ = 0.997; 3SH: 0–3000 ng·L^–1^, *R* ^2^ = 0.983; 3SHA: 0–300 ng·L^–1^, *R* ^2^ = 0.991; 3SHP: 0–560 ng·L^–1^, *R* ^2^ = 0.995; 3SHB: 0–570 ng·L^–1^, *R* ^2^ = 0.955). Selected concentration levels of the derivatized thiols 3SHB and 3SHP are shown in Figure 3. LOD (limit of detection) values were determined as follows: 4MSP (6 ng·L^–1^), 3SH (128 ng·L^–1^), 3SHA (13 ng·L^–1^), 3SHP (12 ng·L^–1^), 3SHB (26 ng·L^–1^); LOQ (limit of quantification): 4MSP (21 ng·L^–1^), 3SH (424 ng·L^–1^), 3SHA (44 ng·L^–1^), 3SHP (39 ng·L^–1^), 3SHB (87 ng·L^–1^).^25^ Agilent MassHunter Workstation Software (version B.08.00) was used to analyze the data.

### NMR

The ^1^H and ^13^C NMR spectra were recorded using a Bruker Avance III NMR spectrometer at 400 and 100 MHz, respectively, at 300 K in CDCl_3_ (99.8 atom % D, containing 0.1% TMS).

### Sensory Analysis: Determination of Detection and Recognition Thresholds of Polyfunctional Thiols in Water

The odor thresholds of 3SH, 3SHA, 3SHP, and 3SHB were determined in ultrapure degassed water. Ethanolic stock solutions of the respective thiols were added to produce ten aqueous solutions of each type of thiol for the sensory analysis (Supporting Information Table S2). The total ethanol concentration in all samples was adjusted to 79 mg·L^–1^ in order to avoid matrix effects. As the odor threshold of ethanol in water is 760 mg·L^–1^, the ethanol concentration used should have no impact on the obtained odor threshold values.^26^

Odor threshold values were determined using the threshold determination test by a trained panel of in total 40 participants in the age range of 20–75 years (average 45.2 years, 35% male/65% female) within two sessions organized over 2 days.^27^ All participants, recruited from (former) employees and students of Hochschule Geisenheim University (Geisenheim, Germany), were members of a trained sensory panel that had completed several weeks of instruction in wine aroma assessment, and most panelists participate in the weekly sensory sessions conducted at the Department of Enology. The sensory evaluations took place in a well-illuminated and odorless sensory analysis room (Department of Enology, Hochschule Geisenheim University). Samples as well as the sensory room were both kept at a temperature of 20 °C. The sensory study was carried out in accordance with the internal ethical and methodological guidelines for sensory analysis established by the Department of Enology (Hochschule Geisenheim University).

Each sensory session included four sensory tests that separately determined sensory thresholds for one of four thiols: 3SH, 3SHA, 3SHP, and 3SHB. During each test, ten wine-tasting glasses (ISO 3591) were provided to each panelist filled with approximately 35 mL of one of the sample solutions (Supporting Information Table S2). The sample in glass 1 was always a control sample (nonthiol-spiked water with ethanol). The panelists were informed that each subsequent glass contained an aqueous sample with a thiol content equal or higher than the glass before. The panelists were asked to identify individually detection and recognition thresholds in each test. In addition, each participant was asked to indicate an olfactory impression (odor descriptors) in each test after reaching the recognition thresholds.

During the test, the participants evaluated the samples orthonasally in ascending order and were not permitted to switch back to previous samples. This precaution was made in case the panelists adapted to the higher thiol concentration and their evaluation of earlier samples changed.

### Statistical and Data Analysis

All fermentations were carried out in triplicate. ETP-derivatizations and measurements of thiols in commercial wines were also performed in triplicate. Visualization of the results for Figure S13 (Supporting Information) was performed using the packages ggplot2 (version 3.5.1), ggthemes (version 5.1.0) and patchwork (version 1.3.0) in R (version 4.4.2) with RStudio (version 2024.12.0). Analysis of variance (ANOVA) and Tukey post hoc tests were performed using the R package multcompView (version 0.1–10). To assess the relationship between the concentration of quantified thiols across all Sauvignon blanc samples, Spearman’s rank correlation coefficients and corresponding *p*-values were calculated and visualized (Supporting Information, Figure S16) using ggplot2 and Hmisc (version 5.2–4) package in R. Geometric mean values of the sensory evaluation were calculated using Microsoft Office.

## Results and Discussion

Preliminary experiments with the addition of various fatty acids and alcohols resulted in a higher formation of the respective esters during fermentation with *S. cerevisiae* VIN13 and VIN13­[tnaA_ATF1] (Supporting Information Figures S13–S15). A comparison with control samples without yeast addition showed that the formation of esters resulted from enzymatic processes during yeast metabolism,^28^ as no chemical esterification could be determined.

### Identification of 3-Sulfanylhexyl Propionate and 3-Sulfanylhexyl Butyrate

Since thiols are highly potent aroma compounds that have a significant impact on the varietal aroma of, for example, Sauvignon blanc wines, it was considered whether thiols that also contain a hydroxyl group could be converted into propionates. For this experiment, 3-sulfanylhexan-1-ol (3SH), known for its grapefruit/passionfruit odor in Sauvignon blanc wines, was used. To increase the probability of detecting potential esters of 3SH, fermentations were conducted in Müller-Thurgau must with the addition of relatively high concentrations of 3SH (500 μg·L^–1^) and propionic acid (100 mg·L^–1^). These fermentations were carried out using the genetically modified *S. cerevisiae* strain VIN13­[tnaA-ATF1] (Supporting Information Table S1), which was selected and constructed based on previous studies.^22^ Due to the overexpression of its native alcohol acetyltransferase encoding genes *ATF1*, this strain was expected to achieve a higher esterification of 3SH to 3SHA.^22^ Also based on previous assumptions, describing that Atf1p might show affinity to propionyl-CoA, a higher formation of propionates could be expected.^21^ Subsequent SPME-GC-MS measurements of the fermentation products showed an unknown peak with RT = 36.98 min. To verify whether this was the propionate of 3SH, 3-sulfanylhexylpropionate (3SHP) was synthesized and measured with the same method. The peak obtained showed the same mass spectra and the same retention time as the unknown peak in the Müller-Thurgau fermentation (Figure 1).

**Figure 1:**
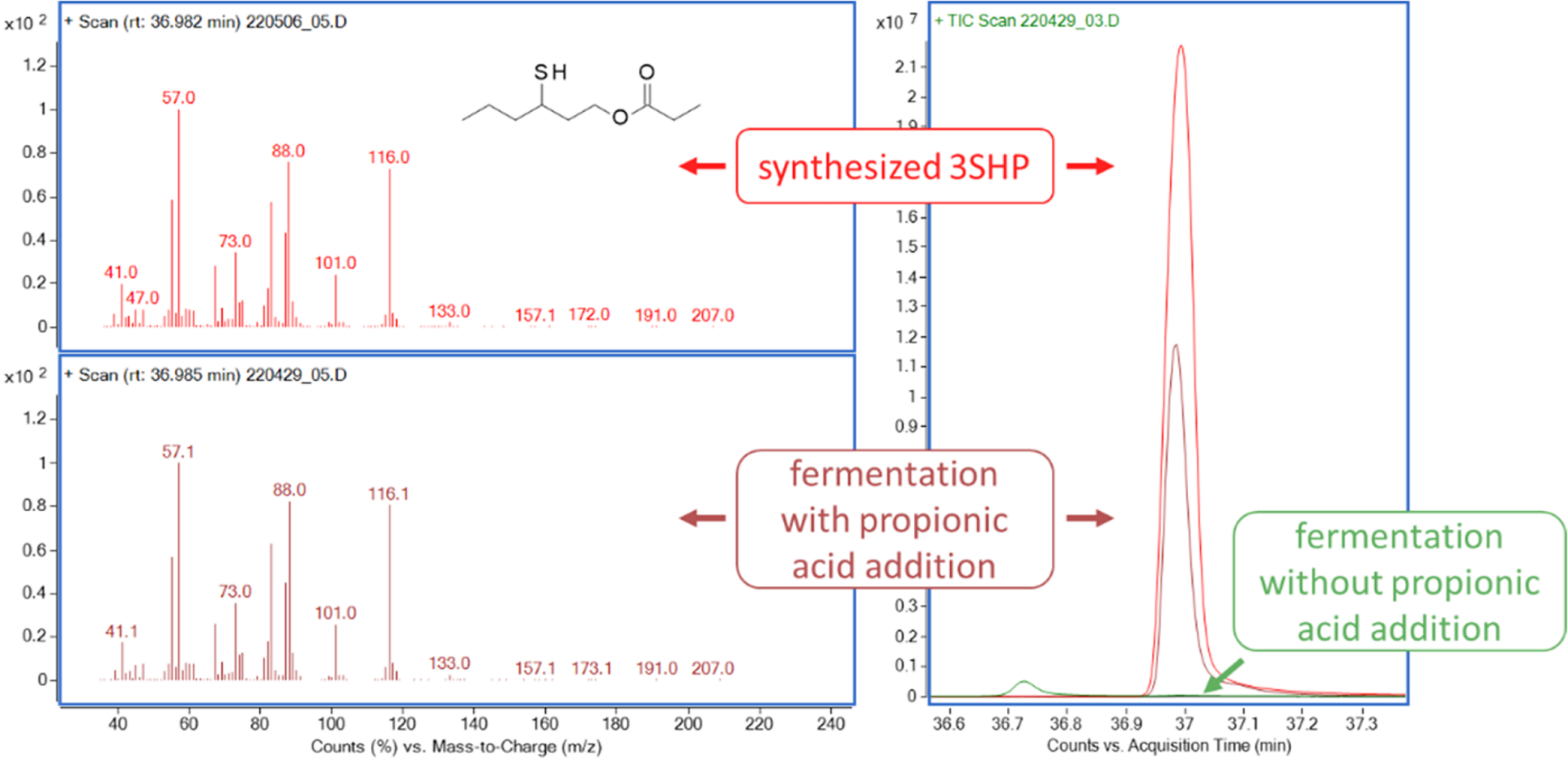
Mass spectra and overlay of chromatograms of synthesized 3-sulfanylhexyl propionate (3SHP) standard and the fermentation sample with propionic acid addition containing the unknown peak.

To verify the hypothesis of increased propionate formation due to the genetic modification of the yeast, comparative fermentations using VIN13 and the strain VIN13­[tnaA-ATF1] were performed (Supporting Information, Figure S13). It was found that, as expected, significantly higher acetate concentrations were obtained in the fermentation with the genetically modified yeast. This is also consistent with previous results.^22^ However, no higher propionate concentrations were detected in the fermentations with GMO compared to non-GMO. This is in accordance with observations from other studies that tested Atf1p against other acyl-CoAs (C_3_, C_4_, C_5_, C_6_, C_8_, C_10_, C_12_) and confirmed that the alcohol acyltransferase activity of Atf1p is promising for alcohols, but very specific for acetyl-CoA.^29^

In addition to the fermentation with propionic acid, butyric acid (100 mg·L^–1^) was added in a further experiment. In this trial, a new peak (RT = 39.28 min) was observed. This peak was identified as 3-sulfanylhexyl butyrate (3SHB) using the NIST 14 database (score 92.9). To verify this peak, 3SHB was synthesized and measured using the same SPME-GC-MS method (Figure 2).

**Figure 2:**
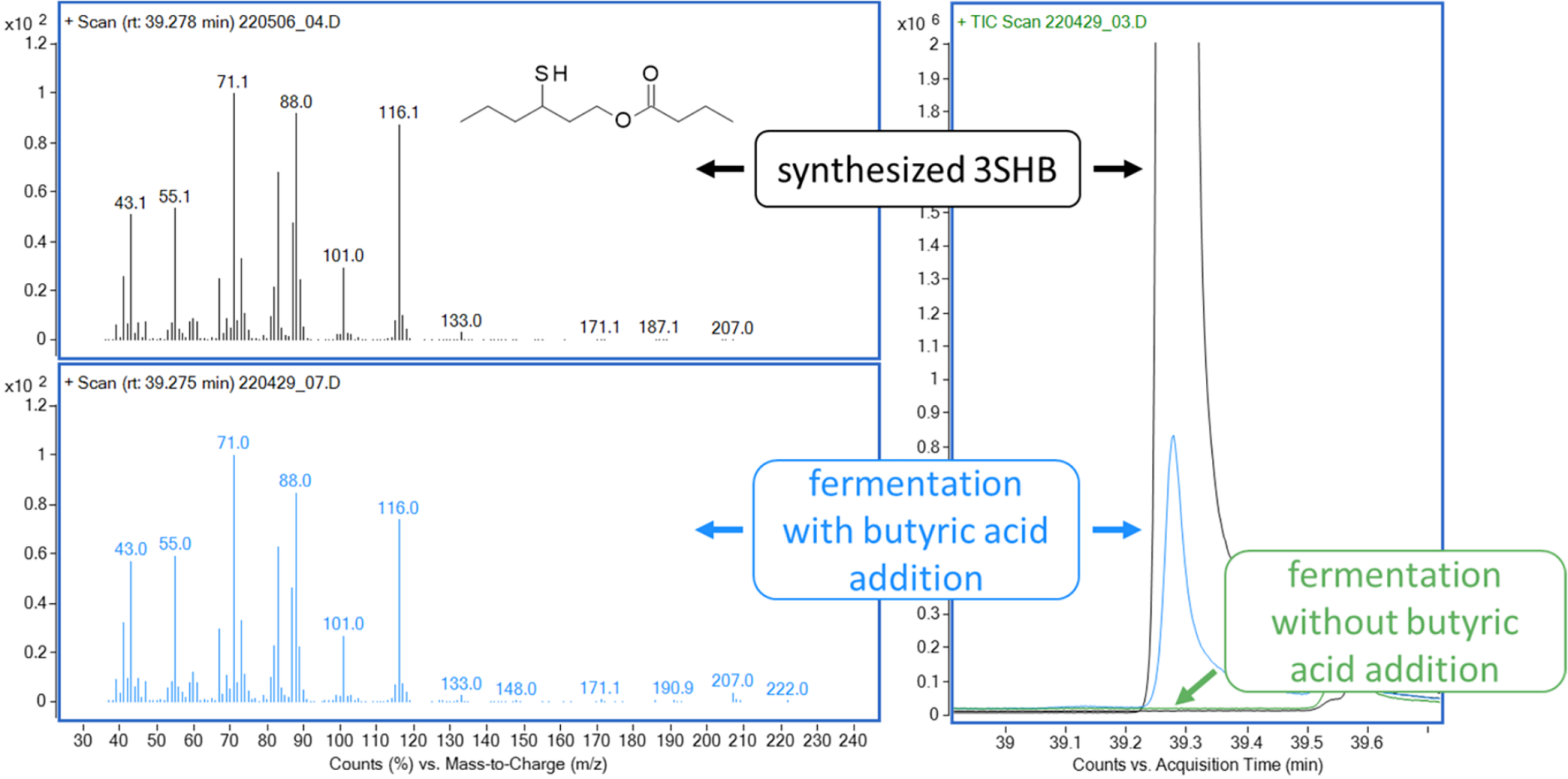
Mass spectra and overlay of chromatograms of synthesized 3-sulfanylhexyl butyrate (3SHB) standard and the fermentation sample with butyric acid addition containing the unknown peak.

Based on the measurement, it was possible to determine that the identified compound was 3SHB, since the mass spectra as well as the retention times were identical. In a comparative fermentation with the addition of the same 3SH concentration but without propionic acid or butyric acid, no detectable peaks for 3SHP and 3SHB were observed in full-scan GC-MS measurements.

### Quantification of 3-Sulfanylhexyl Propionate (3SHP) and 3-Sulfanylhexyl Butyrate (3SHB) in Commercial White Wines

To verify the presence of 3SHP and 3SHB in wines, commercial Scheurebe, Sauvignon blanc and Verdejo wines from different regions were obtained, as 3SH and the acetate esters 3SHA have already been described in these grape varieties.^30^ Wines from various origins were selected because regional factors such as water availability, sunlight exposure, nitrogen supplementation and temperature conditions can influence the accumulation of thiol precursors.^31–33^ Neither the propionyl nor the butanoyl esters of 3-sulfanylhexan-1-ol have been previously described as wine constituents. In this study, we could quantitate 3SHP and 3SHB for the first time in commercial white wines. 3-Sulfanylhexyl propionate was previously described only in extracts from *Ruta chalepensis* L. and 3-sulfanylhexyl butyrate has been characterized as an aroma compound of passionfruit by several authors.^34–36^

To measure the commercial wines, the previous SPME-GC-MS method could not be used, as concentrations in the lower ng·L^–1^ range were expected. Therefore, GC-MS measurements were performed in SIM mode after prior derivatization with ethyl propiolate (ETP). Mass spectra showing the ETP derivatives of 3SHP and 3SHB as well as the chromatograms of a commercial Sauvignon blanc wine spiked with 3SHP or 3SHB in increasing concentration, are shown in Figure 3. In addition to 3SHP and 3SHB, the thiols 4MSP, 3SH and 3SHA were determined in all wines using the same method (Table 1).

**Figure 3:**
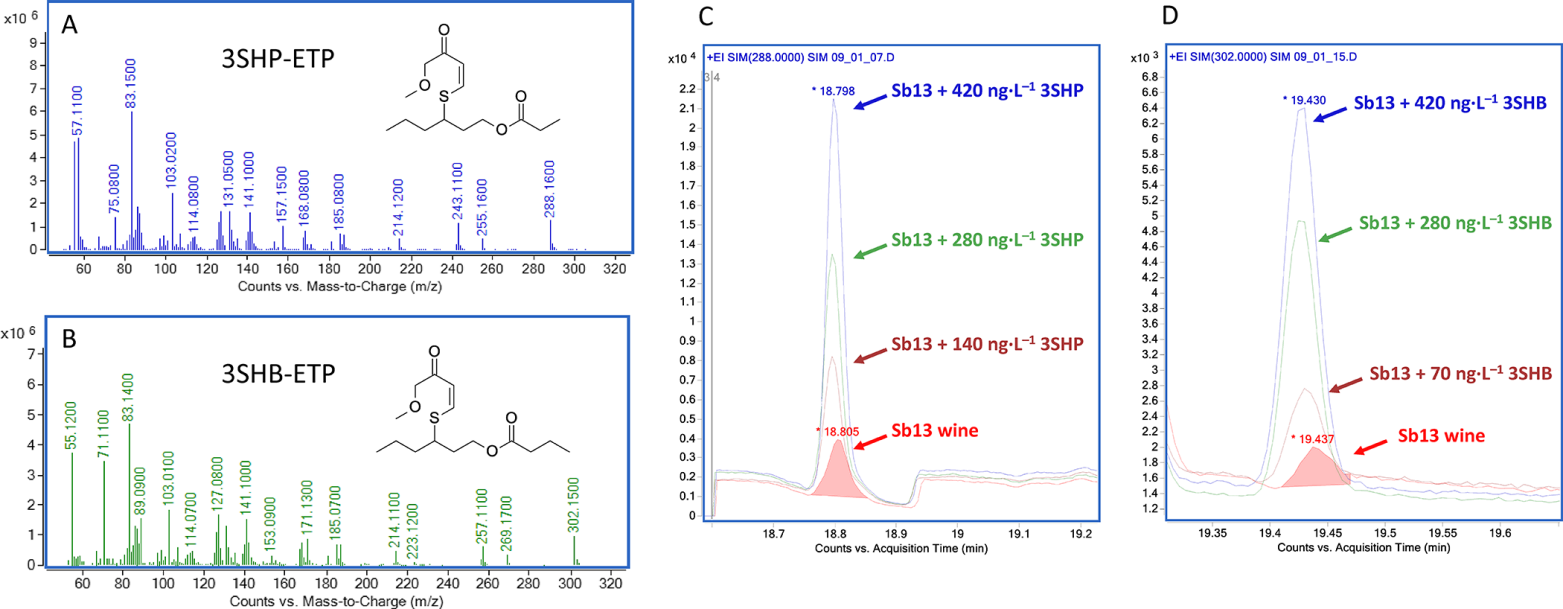
Mass spectra of the ethylpropiolate (ETP) derivatives from (A) 3-sulfanylhexyl propionate (3SHP) and (B) 3-sulfanylhexyl butyrate (3SHB) as well as overlaid selected ion chromatograms of a commercial Sauvignon blanc wine (Sb13) spiked with (C) 3SHP (3SHP-ETP: *m*/*z* 288) and (D) 3SHB (3SHB-ETP: *m*/*z* 302). (A, B) show full-scan mass spectra of derivatized standards recorded during method development to identify ions used for selected ion monitoring (SIM) analysis. (C, D) display SIM chromatograms of wine samples spiked and unspiked with the corresponding thiol.

**Table 1:** Concentration of 4-Methyl-4-sulfanylpentan-2-one (4MSP), 3-Sulfanylhexan-1-ol (3SH), 3-Sulfanylhexyl Acetate (3SHA), 3-Sulfanylhexyl Propionate (3SHP) and 3-Sulfanylhexyl Butyrate (3SHB) in Commercial Scheurebe (Scheu), Sauvignon blanc (Sb) and Verdejo (Ver) Wines (Analyzed in September 2022)^a^,^b^,^c^

				thiol concentration (ng·L^–1^)				
wine	vintage	wine region	country	4MSP	3SH	3SHA	3SHP	3SHB
Scheu1	2021	Franken	Germany	(16 ± 1)^e^	827 ± 70	44 ± 2	(24 ± 7)^e^	(9 ± 2)^d^
Scheu2	2021	Palatinate	Germany	(9 ± 0)^e^	467 ± 28	(33 ± 2)^e^	nq	(8 ± 1)^d^
Scheu3	2021	Rheinhessen	Germany	(8 ± 0)^e^	552 ± 244	(33 ± 1)^e^	63 ± 31	nd
Scheu4	2021	Rheinhessen	Germany	(14 ± 1)^e^	(312 ± 59)^e^	(34 ± 3)^e^	(31 ± 3)^e^	nd
Sb1	2021	Central Valley	Chile	nd	2047 ± 474	(31 ± 1)^e^	87 ± 17	(8 ± 1)^d^
Sb2	2021	Roussillon	France	(16 ± 1)^e^	1871 ± 288	(36 ± 6)^e^	nq	nd
Sb3	2021	Palatinate	Germany	22 ± 2	1802 ± 113	(38 ± 0)^e^	104 ± 6	nq
Sb4	2021	Loire	France	nd	1307 ± 64	54 ± 1	(34 ± 6)^e^	nd
Sb5	2021	Navarra DOP	Spain	nd	1726 ± 745	244 ± 16	nq	(53 ± 4)^e^
Sb6	2020	Elgin	South Africa	nd	(3372 ± 536)^f^	221 ± 18	43 ± 13	nd
Sb7	2021	Rueda DOP	Spain	(12 ± 1)^e^	1182 ± 47	(28 ± 0)^e^	(21 ± 6)^e^	(21 ± 5)^d^
Sb8	2020	Bordeaux	France	27 ± 0	1224 ± 103	(29 ± 5)^e^	52 ± 7	nd
Sb9	2021	Marlborough	New Zealand	29 ± 1	(5416 ± 91)^f^	95 ± 2	83 ± 10	(10 ± 4)^d^
Sb10	2021	Rheinhessen	Germany	(19 ± 1)^e^	1169 ± 206	(37 ± 2)^e^	62 ± 6	nd
Sb11	2009	Rheingau	Germany	nd	840 ± 33	nq	nd	nd
Sb12	2021	Rheingau	Germany	(20 ± 1)^e^	1847 ± 494	(43 ± 2)^e^	53 ± 20	(16 ± 2)^d^
Sb13	2021	Western Cape W.O.	South Africa	nd	1673 ± 176	57 ± 3	46 ± 6	(22 ± 6)^d^
Sb14	2021	Western Cape W.O.	South Africa	nd	850 ± 168	(39 ± 3)^e^	49 ± 6	(18 ± 2)^d^
Sb15	2021	Western Cape W.O.	South Africa	nq	484 ± 100	(33 ± 2)^e^	nq	nq
Ver1	2021	Rueda DOP	Spain	nd	541 ± 69	50 ± 1	(29 ± 2)^e^	nd
Ver2	2021	Castilla y León IGP	Spain	nd	819 ± 208	53 ± 6	nd	nd
Ver3	2021	Rueda DOP	Spain	nd	851 ± 49	(43 ± 1)^e^	(34 ± 16)^e^	nd

a Values are means ± standard deviations from triplicate measurements.

b nd: not detected; nq: not quantifiable due to matrix effects; LOD (limit of detection): 4MSP (6 ng·L^–1^), 3SH (128 ng·L^–1^), 3SHA (13 ng·L^–1^), 3SHP (12 ng·L^–1^), 3SHB (26 ng·L^–1^); LOQ (limit of quantification): 4MSP (21 ng·L^–1^), 3SH (424 ng·L^–1^), 3SHA (44 ng·L^–1^), 3SHP (39 ng·L^–1^), 3SHB (87 ng·L^–1^).

c Values in brackets are estimated concentrations, because they are:

d below LOD,

e below LOQ, or

f above the calibration range.

3SHP was detected in three of four Scheurebe wines, 11 of 15 Sauvignon blanc wines and two of three Verdejo wines tested. Concentrations of up to 104 ng·L^–1^ were determined in Sauvignon blanc wines, which also exhibited the broadest concentration range (21–104 ng·L^–1^). Scheurebe wines showed concentrations of 24–63 ng·L^–1^, while the two Verdejo wines with detectable 3SHP contained 29 and 34 ng·L^–1^, respectively.

3SHB was detected in two of four Scheurebe wines with concentration of 8 and 9 ng·L^–1^, while seven of 15 Sauvignon blanc wines showed levels ranging from 8 to 53 ng·L^–1^. In Verdejo wines, 3SHB could not be detected. However, since only a very small number of Verdejo wines were examined in this study, it cannot be ruled out that 3SHB could also be present in other Verdejo wines. Furthermore, it is possible 3SHB is present in the investigated Verdejo wines in concentrations not measurable with our methods.

Since propionic acid can occur in wines in significantly higher concentrations (up to 100 mg·L^–1^) than butyric acid (0.4–5 mg·L^–1^),^20^ this can explain the higher concentration of the measured 3SHP compared to 3SHB. Preliminary fermentation experiments support this explanation, as the addition of propionic acid increased propionate formation compared to fermentations without acid supplementation (Supporting Information Figure S14). Experiments using different short- (propionic acid, and butanoic acid) and medium-chain fatty acids (hexanoic acid, and octanoic acid) were conducted to investigate the formation of further esters (Supporting Information Figure S15). Hexan-1-ol was used as a model alcohol due to its chemical stability and structural similarity to 3-sulfanylhexan-1-ol, indicating that esterification mechanisms are comparable. In these experiments, the respective esters of hexan-1-ol were found in higher concentrations only when the corresponding fatty acid was added, confirming that substrate availability strongly influences ester formation via yeast metabolism. This is consistent with the role of fatty acids as precursors for acyl-CoA intermediates in yeast ester synthesis.^37,38^

The fact that 3SHB and 3SHP were found in a large number of samples of Sauvignon blanc and Scheurebe wines, and 3SHP in Verdejo wines, suggests that these compounds may also be present in wines from other grape varieties that are known to contain polyfunctional thiols.

To evaluate the correlation between the concentrations of volatile thiols (4MSP, 3SH, 3SHA, 3SHP and 3SHB) in Sauvignon blanc, Spearman’s rank correlation analysis was performed (Supporting Information Figure S16). The data shows a moderate positive correlation between 4MSP and 3SHP (*r* = 0.57, *p* ≤ 0.05), 3SH and 3SHA (*r* = 0.57, *p* ≤ 0.001), 3SH and 4MSP (*r* = 0.53, *p* ≤ 0.05), as well as between 3SH and 3SHP (*r* = 0.37, *p* ≤ 0.05). In contrast, strong significant negative correlations were found between 3SHB and both 4MSP (*r* = −0.75, *p* ≤ 0.05) and 3SHP (*r* = −0.75, *p* ≤ 0.001). Moderate negative correlations were found between 3SHB and 3SH (*r* = −0.48, *p* ≤ 0.05). The positive correlation between 3SH and its esters (3SHP and 3SHA) is consistent with the fact that 3SH acts as a precursor for these compounds.

### Sensory Evaluation of 3SHP and 3SHB

The threshold values were calculated in two ways, as outlined in the method description.^39^ One approach involved finding the lowest value indicated by 50% of the tasters. The other procedure involved calculating the geometric mean from all panelists’ responses. During the sensory characterization, all participants were asked to mention their odor impressions of each thiol. Table 2 presents the three most frequently mentioned descriptors.

**Table 2:** Detection Threshold, Recognition Threshold and Odor Description of 3-Sulfanylhexan-1-ol and Its Acetate (3SHA), Propionate (3SHP) and Butyrate (3SHB) Based on our Sensory Tests and Data from the Literature

thiol	odor description	literature	detection threshold (ng·L^–1^)	recognition threshold (ng·L^–1^)	literature (ng·L^–1^)
3-sulfanylhexan-1-ol (3SH)	grapefruit, passionfruit, tropical fruits	grapefruit, passionfruit^40^	50% panelists: 16	50% panelists: 33	water:17 ^40^ ^,^ ^c^
			geometric mean: 17.0	geometric mean: 36.6	model wine^a^: 60 ^40^ ^,^ ^c^
3-sulfanylhexyl acetate (3SHA)	passionfruit, grapefruit, tropical fruits	box tree, passionfruit^40^	50% panelists: 0.8	50% panelists: 2.4	water: 2.3,^40^ ^,^ ^c^
			geometric mean: 0.8	geometric mean: 2.3	model wine^a^: 4.2,^40^ ^,^ ^c^
3-sulfanylhexyl propionate (3SHP)	passionfruit, grapefruit, tropical fruits	sulfurous, phosphorus^34^ ^,^ ^b^	50% panelists: 38.4	50% panelists: 76.8	-
			geometric mean: 45.7	geometric mean: 116.0	
3-sulfanylhexyl butyrate (3SHB)	passionfruit, grapefruit, tropical fruits	sulfurous, passionfruit, fruity, tropical^36^	50% panelists: 76.7	50% panelists: 299.4	-
			geometric mean: 98.4	geometric mean: 223.1	

a Threshold determined in water/ethanol 12% (v/v) + 5 g·L^–1^ tartaric acid, pH = 3.5

b Odor descriptors of the undiluted racemic reference compound.

c Detection threshold, 50% panelists

Odor detection and recognition thresholds for the four thiols were summarized as follows: The detection threshold of 3SH was identified at 16–17 ng·L^–1^, which is consistent with the literature value of 17 ng·L^–1^ for the odor threshold in water.^40^ The recognition threshold for 3SH was determined to be between 33 and 37 ng·L^–1^. For 3SHA, a detection threshold of 0.8 ng·L^–1^ and a recognition threshold of 2.3–2.4 ng·L^–1^ were measured. The determined recognition threshold aligns with the published odor threshold for 3SHA in water, which is 2.3 ng·L^–1^.^40^ 3SHP exhibited detection thresholds ranging from 38 to 46 ng·L^–1^ and a recognition threshold of 77–116 ng·L^–1^, whereas for 3SHB detection thresholds were approximately 77–98 ng·L^–1^, with a recognition threshold of about 223–299 ng·L^–1^. Although the odor thresholds of 3SHP and 3SHB are higher than those for 3SH or 3SHA, they remain within the ng·L^–1^ range, confirming their high odor potency. In sensory evaluation, 3SHP and 3SHB were primarily described with passionfruit, grapefruit, and tropical fruit notes, in agreement with the odor descriptors previously reported for 3SHB.^36^ To evaluate the impact of the newly identified thiols 3SHP and 3SHB, the measured concentrations (Table 1) were compared with the odor threshold values (Table 2). It was found that the concentrations of 3SHP in the analyzed commercial wines were below or slightly above the recognition threshold in water, while all measured concentrations of 3SHB are below the detection threshold in water. Odor thresholds determined in water are generally lower than those measured in hydroalcoholic matrices or wine, implying that 3SHB and 3SHP may have a limited impact on wine aroma perception. At the same time, their structural and sensory similarity to 3SH and 3SHA suggests that both thiols (3SHP and 3SHB) may have an enhancing effect on the grapefruit/passionfruit/tropical fruit aroma of the respective wine, as already reported for other aroma compound mixtures at subthreshold levels.^41^

In the analytical measurements, the limits of detection (LOD) and quantification (LOQ) for 3SHP were 12 ng·L^–1^ and 39 ng·L^–1^, respectively, and for 3SHB 26 ng·L^–1^ and 87 ng·L^–1^. These values are below the corresponding recognition thresholds and close to the detection thresholds in water. This indicates that the current GC–MS method provides sufficient sensitivity for determining concentrations relevant to odor perception. However, the use of more advanced techniques, such as tandem mass spectrometry, could allow the detection of 3SHP and 3SHB at or below their recognition thresholds if required for future studies. It is noticeable that all compounds (3SH, 3SHA, 3SHP, and 3SHB) were associated with grapefruit, passionfruit, and tropical fruit aromas. This also aligns with the literature references regarding the odor impressions of 3SH and 3SHA,^40^ which were similarly evaluated with these scents. A study by Polster and Schieberle evaluated the odor impressions of 49 sulfanylalkanols and supports this.^42^ These authors found that many of the investigated 3-sulfanyl-3-methylalkan-1-ols, 1-sulfanyl-2-methylalkan-3-ols, 2-sulfanylalkan-1-ols, 4-sulfanylalkan-2-ols, and 3-sulfanylalkan-1-ols exhibit a grapefruit odor.^42^ Furthermore, it is noteworthy that the odor threshold increases with increasing chain length. This also corresponds with findings from the literature, as sensory characterizations of 3-sulfanylalkan-1-ols showed that thiols with a carbon chain length of 6–7 have the lowest odor threshold, while the odor thresholds continuously increase with both longer and shorter carbon chain lengths.^42^

Besides the concentrations found in Sauvignon blanc, Scheurebe and Verdejo, it can be assumed that 3SHB and 3SHP also occur in wines from other grape varieties associated with 3SH. Moreover, these compounds may be present at higher concentrations in wines made from botrytized Sauvignon blanc must due to the higher concentration of cysteinylated 3SH precursors compared to nonbotrytized samples,^43^ and the greater consumption of cysteinylated precursors by the yeast during fermentation.^18^

Furthermore, the concentration of 3SHP and 3SHB could be increased through fermentation strategies. For example, the application of nonconventional yeasts as well as selected lactic and acetic acid bacteria can increase the formation of key aroma compounds.^44^ A further study showed that fermentations performed with *Torulaspora delbrueckii* resulted in significantly higher concentrations of ethyl propionate, especially when sequential fermentations with *T. delbrueckii* and *S. cerevisiae* were conducted.^45^ Although the formation of other propionate esters was not quantitated in this study, *T. delbrueckii* may also influence the production of 3SHP. The formation of 3SHB could also be positively influenced by *T. delbrueckii* fermentations, as studies have found an increased formation of other butyrates such as ethyl 2-methylbutyrate.^46^ Furthermore, 3SH release is subject to nitrogen catabolite repression, resulting in higher 3SH concentrations at lower yeast-assimilable nitrogen (YAN) levels, while ester synthesis increases with higher YAN availability due to enhanced yeast growth and acyltransferase activity.^47–49^

It is also possible that wines analyzed immediately after fermentation may contain higher concentrations of 3SHB and 3SHP, as these aroma compounds may undergo hydrolysis into the corresponding acids and 3SH during storage. Measurements of the pure synthesized reference compounds showed that within two years 70% of the 3SHB stored at −20 °C hydrolyzed into butyric acid and 3SH. At the same time, under the same storage conditions, 30% of the synthesized 3SHP hydrolyzed. Given that lower pH,^20^ extended storage durations and higher storage temperatures promote hydrolysis,^50^ and considering that these variables are unknown for the wines investigated in this study, it is possible that initially higher concentrations of 3SHP and 3SHB were present.

All three esters 3SHA, 3SHP, and 3SHB are formed from 3SH. The conversion of 3SH to 3SHA yields a more potent aroma compound, thanks to 3SHA’s very low detection and recognition threshold. As a result, the produced 3SHA increases the overall tropical fruit aroma intensity of the wine relative to 3SH. In contrast, the formation of 3SHP and 3SHB from 3SH yields thiol esters with considerably higher odor thresholds than 3SHA and 3SH. Therefore, converting 3SH into 3SHP and 3SHB would reduce the concentration of the more powerful 3SH and the aroma intensity of the resulting wine. However, due to their hydrolysis, 3SHP and 3SHB could act as reservoirs for 3SH; nevertheless, given their low concentration relative to typical 3SH levels, their contribution to aroma changes is expected to be minimal. As a result, the balance between 3SH and its esterified derivatives is an important factor that determines the sensory effect of volatile thiols in wine.

In summary, two new thiols, 3SHP and 3SHB, were described in wine for the first time. 3SHP was detected in commercial wines above the determined detection threshold and in three wines in the range of the perception threshold. Due to its grapefruit/passionfruit aroma, 3SHP could contribute positively to the overall impression of wines and could complement the impression of 3SH and 3SHA. Although 3SHB could only be detected below the odor threshold in the wines tested, it has not yet been determined whether 3SHB has a supporting effect on the grapefruit aroma. The possibility of odor-enhancing effects still needs to be investigated in further studies. In addition, sensory relevant concentrations of 3SHP and 3SHB could occur in wines of other grape varieties and vintages.

In line with the hypothesis of Waterhouse et al. that thousands of esters can be present in wine in minute concentrations,^20^ this study also showed that measurable ester concentrations were formed during fermentations of musts enriched with the corresponding alcohols and carboxylic acids. It can therefore be assumed that, in addition to 3SHP and 3SHB, further esters of 3SH with other fatty acids, as well as many other yet to be identified esters of other alcohols (with and without a thiol group), could also be present in the wine. Based on previous literature and our results for 3SHP and 3SHB,^42^ it can also be assumed that further 3SH esters formed with fatty acids of increasing chain length may have increasing odor thresholds and become less sensorially relevant. These newly identified esters (3SHP and 3SHB) are chiral, and further studies could investigate potential differences in thresholds and odor qualities among their enantiomers. To identify and characterize these compounds, understand their contribution to wine aroma, and clarify their biochemical and sensory relevance, further studies combining more complex analytical methods with biochemical and sensory investigations are required.

## Supplementary Material



## Notes

Chemicals and biological reagents in this study; generation of the yeast strain VIN13­[tnaA_ATF1]; GC-MS chromatograms and mass spectra of 3-sulfanylhexyl butyrate and 3-sulfanylhexyl propionate; ^1^H NMR, ^13^C NMR and DEPT-135 spectra of 3-sulfanylhexyl butyrate and 3-sulfanylhexyl propionate; table of thiol concentrations for sensory determination of odor thresholds; results of fermentation trials with GMO; results of preliminary fermentation trials with fatty acid addition; Spearman correlation (PDF)

## ⊥

BRAIN Biotech AG, Darmstädter Straße 34–36, 64673 Zwingenberg, Germany

## Abbreviations

ANOVA: analysis of variance
DCM: dichloromethane
EI: electron impact
ETP: ethyl propiolate
GC-MS: gas chromatography–mass spectrometry
HS-SPME: headspace solid-phase microextraction
4MMB: 4-methoxy-2-methyl-2-butanethiol
4MSP: 4-methyl-4-sulfanylpentan-2-one
MT: Müller-Thurgau
NMR: nuclear magnetic resonance
RT: retention time
Sb: Sauvignon blanc
Scheu: Scheurebe
3SH: 3-sulfanylhexan-1-ol
3SHA: 3-sulfanylhexyl acetate
3SHB: 3-sulfanylhexyl butyrate
3SHP: 3-sulfanylhexyl propionate
SIM: selected ion monitoring
Ver: Verdejo
YAN: yeast available nitrogen
