# Supplementary material for: Identification and Sensory Evaluation of 3‑Sulfanylhexyl Propionate and 3‑Sulfanylhexyl Butyrate in Wine
Source: J Agric Food Chem. 2026 Jan 30;74(5):4693–702. doi: 10.1021/acs.jafc.5c12053 (PMC12903898; doi:10.1021/acs.jafc.5c12053)
Supplement: Supplementary file 1 [file jf5c12053_si_001.pdf]

Supporting Information

# Identification and Sensory Evaluation of 3-Sulfanylhexyl Propionate and 3-Sulfanylhexyl Butyrate in Wine

Florian Kiene [1*], Niël van Wyk [1,2#], Claus Patz [3], Andrii Tarasov [4], Vicky Bäumer [3], Christoph Schüßler [4], Rainer Jung [4], Christian von Wallbrunn [1], Isak S. Pretorius [2], and Doris Rauhut [1]

[1] Department of Microbiology and Biochemistry, Hochschule Geisenheim University, Von-Lade-Straße 1, 65366 Geisenheim, Germany

[2] ARC Centre of Excellence in Synthetic Biology, Department of Molecular Sciences, Macquarie University, Sydney, NSW 2113, Australia

[3] Department of Beverage Research, Hochschule Geisenheim University, Von-Lade-Straße 1, 65366 Geisenheim, Germany

[4] Department of Enology, Hochschule Geisenheim University, Von-Lade-Straße 1, 65366 Geisenheim, Germany

[#] Present address: BRAIN Biotech AG, Darmstädter Straße 34-36, 64673 Zwingenberg, Germany

* Corresponding author: florian.kiene@hs-gm.de

Table of Contents


1. Chemicals and biological reagents ....................2
2. Generation of the yeast strain VIN13[tnaA_ATF1] ....................3
3. GC-MS chromatograms ....................5
 3.1 3-Sulfanylhexyl butyrate ....................5
 3.2 3-Sulfanylhexyl propionate ....................6
4. Mass spectra ....................7
 4.1 3-Sulfanylhexyl butyrate ....................7
 4.2 3-Sulfanylhexyl propionate ....................7
5. NMR-spectra ....................8
 5.1 3-Sulfanylhexyl butyrate ....................8
 5.2 3-Sulfanylhexyl propionate ....................9
6. Sensory evaluation ....................11
7. Fermentation trials with GMO ....................11
8. Preliminary fermentation trials with fatty acid addition ....................12
9. Spearman Correlation ....................13
10. References ....................13

## 1. Chemicals and biological reagents

Ethanol (99.97%, CAS: 64-17-5), ethyl acetate (≥99.8%, CAS: 141-78-6) and yeast extract (CAS: 8013-01-2) were ordered from Avantor Inc. (Radnor, PA, USA), dichloromethane (≥99.5%, CAS: 75-09-2), n-hexane (≥99%, CAS: 110-54-3), methanol (≥99.9%, CAS: 67-56-1), peptone ex casein (CAS: 91079-40-2), sodium chloride (≥99.5%, CAS: 7647-14-5), sodium hydrogencarbonate (≥99.5%, CAS: 144-55-8), sodium hydroxide (≥99%, CAS: 1310-73-2) and sodium sulfate (≥99%, CAS: 7757-82-6) were bought from Carl Roth GmbH + Co. KG (Karlsruhe, Germany), butyryl chloride (99%, CAS: 141-75-3), D-glucose (Laboraotry reagent grade, CAS: 50-99-7), iodine (≥99%, CAS: 7553-56-2), 4-methyl-4-sulfanylpentan-2-one (98%, CAS: 19872-52-7), propionyl chloride (98%, CAS: 79-03-8) and 3-sulfanylhexan-1-ol (96%, CAS: 51755-83-0) were bought from Thermo Fisher Scientific Inc. (Waltham, MA, USA), and the following chemicals were purchased from Merck KGaA (Darmstadt, Germany): butyric acid (≥99%, CAS: 107-92-6), chloroform-$d_3$ (≥99%, 99.8 atom % D, CAS: 865-49-6, containing 0.1 % TMS), ethyl propiolate (99%, CAS: 623-47-2), hexanoic acid (≥99%, CAS: 142-62-1), 1-hexanol (≥99.9%, CAS: 111-27-3), 4-methoxy-2-methyl-2-butanthiol (≥98%, CAS: 94087-83-9), 3-methyl-1-butanol (≥99%, CAS: 123-51-3), octanoic acid (≥99%, CAS: 124-07-2), pentan-1-ol (≥99.9%, CAS: 71-41-0), 2-phenylethanol (≥99%, CAS: 123-51-3), propionic acid (≥99.5%, CAS: 79-09-4), 3-sulfanylhexyl acetate (≥98%, CAS: 136954-20-6).

## 2. Generation of the yeast strain VIN13[tnaA_ATF1]

To achieve overexpression of the *ATF1* open reading frame (ORF), a CRISPR-Cas9 approach was employed to replace it native promoter with the constitutive *TEF1* promoter from *S. cerevisiae*. A repair template was designed, consisting of the *TEF1* promoter flanked by 500 bp regions of the *ATF1* ORF and 500 bp upstream of *ATF1*'s predicted promoter. This template was synthesized by Genscript (Piscataway, NJ, USA). The Cas9 enzyme along with guide RNAs were introduced into the yeast cells using the previously constructed pCas9-Hyg plasmid.[1] A guide targeting the promoter region of *ATF1*, upstream of its PAM site, was incorporated using primers with overhangs complementary to the guide RNA sequence.

The *tnaA* gene (1416 bp) from *E. coli*, encoding a tryptophanase with known cysteine-β-lyase activity, was codon-optimized by Genscript for expression in *S. cerevisiae*. This gene was cloned into a pUC57 plasmid flanked by PacI and AscI restriction sites (New England Biolabs, MA). Using these sites, the *tnaA* gene was excised and ligated into a linearized pBKD plasmid using T4 DNA ligase (Thermo Fisher Scientific, MA), resulting in the plasmid pBKD_tnaA. The pBKD vector is a shuttle vector compatible with *E. coli* and *S. cerevisiae*, containing a geneticin resistance marker (KanMX), a *PGK1* promoter and terminator, separated by PacI and AscI sites, and flanked by delta sequences derived from the Ty1 retrotransposon of *S. cerevisiae*. These delta sequences facilitated stable genomic integration of pBKD_tnaA.

Yeast transformations followed previously established protocols. [2] Initially, about 100 ng of linearized pCas9-Hyg and 200 ng of the repair template were used to transform the VIN13 cells via electroporation. The cells were then plated on YEPD agar (10g·L$^{-1}$ yeast extract, 20 g·L$^{-1}$ peptone, 20 g·L$^{-1}$ glucose, 20 g·L$^{-1}$ agar) containing 200 µg/mL hygromycin and incubated at 30 °C for two days. Colonies were streaked onto nonselective YEPD plates, and genomic DNA was extracted using a previously published method [3] to verify promoter replacement via PCR (see primers in **Table S1**). Colonies with confirmed promoter exchanges, were subsequently transformed with the linearized pBKD_tnaA plasmid, cut with XhoI. These transformants were selected on YEPD plates containing 100 µg·mL$^{-1}$ geneticin and screened for tnaA insertion via PCR using primers listed in **Table S1** generating the strain VIN13[tnaA_ATF1].

**Table S1.** Primers used to construct and verify the strain used in this study.

| Purpose | Oligo sequence |
|---|---|
| to amplify repair cassette TEF1p_ATF1 from synthesized construct | forward: 5′-GTTATACCACTGTAGGAAGAG-3′<br>reverse: 5′-GGTCCTGTTGGTCCAAAGTAAG-3 |
| guide-RNA primers targeting the native *ATF1* promoter (guide underlined) | forward: 5′-<u>CGAAGAAATGCAAAGAAGTA</u>GTTTTAGAGCTAGAAATAGCAAGTTA-3′<br>reverse: 5′-<u>TACTTCTTTGCATTTCTTCG</u>GATCATTTATCTTTCACTGCG-3′ |
| Verification of native promoter exchange to TEF1p | forward: 5′-GTTATACCACTGTAGGAAGAG-3′<br>reverse: 5′-CTAAGGGCCTAAAAGGAGAG-3′ |
| *tnaA* cloning and verification primers (restriction sites underlined) | forward: 5′- CCC<u>TTAATTAA</u>ATGGAAAACTTTAAACACTTG-3′<br>reverse: 5′-AAA<u>GGCGCGCC</u>TCAGACTTCTTTCAACTTAG-3′ |

# 3. GC-MS chromatograms

## 3.1 3-Sulfanylhexyl butyrate

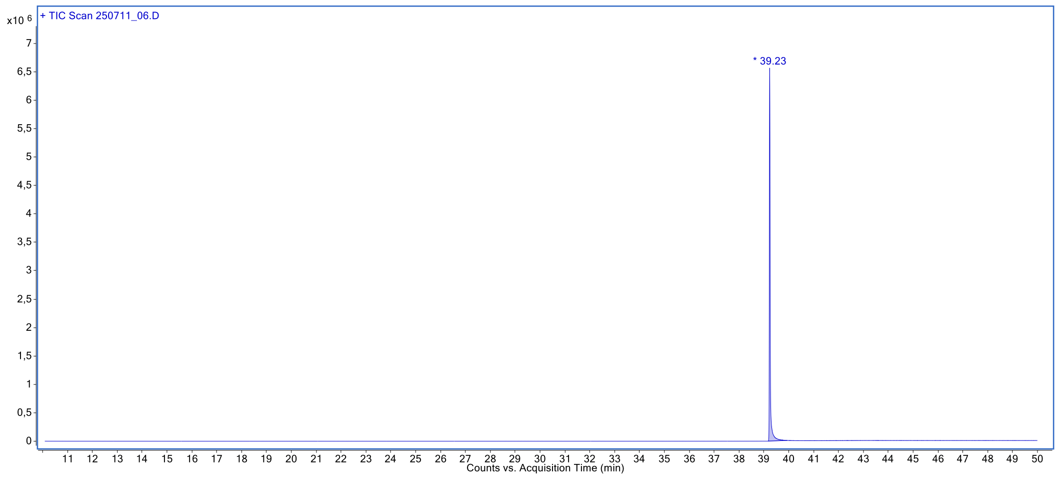


**Figure S1.** HS-SPME-GC-MS chromatogram of 3-sulfanylhexyl butyrate. Method: 40 °C, 4 min; 5 °C/min → 210 °C, 0 min; 20 °C/min → 240 °C, 10.5 min; 1.2 mL/min helium (constant flow); column: 60 m × 0.25 mm ID, 1.0 µm $d_f$, Rxi-5Sil MS (Restek GmbH, Bad Homburg, Germany).

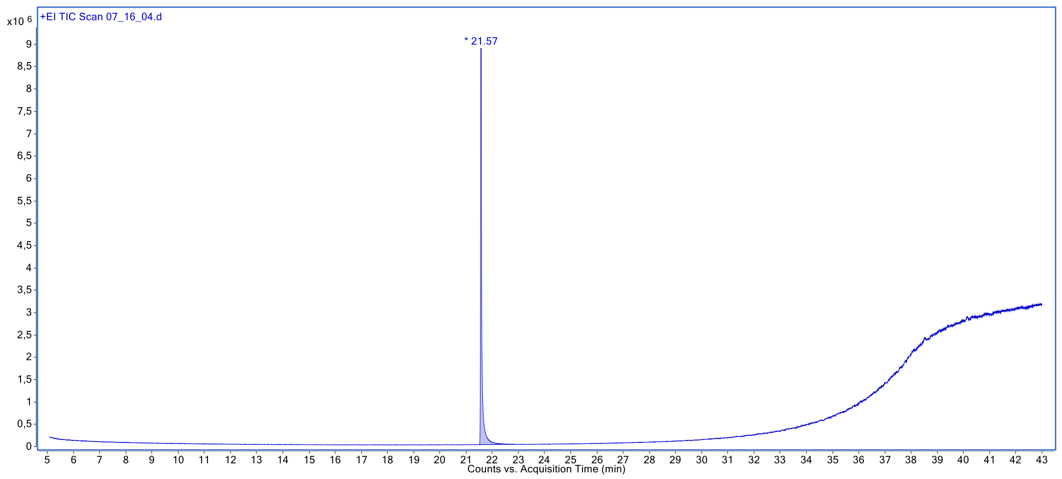


**Figure S2.** GC-MS chromatogram of 3-sulfanylhexyl butyrate in DCM. Method: 40 °C, 3 min; 6 °C/min → 250 °C, 5 min; 1.2 mL/min helium (constant flow); column: 30 m × 0.25 mm ID × 0.25 µm $d_f$, Stabilwax-DA (Restek GmbH, Bad Homburg, Germany).

## 3.1 3-Sulfanylhexyl propionate

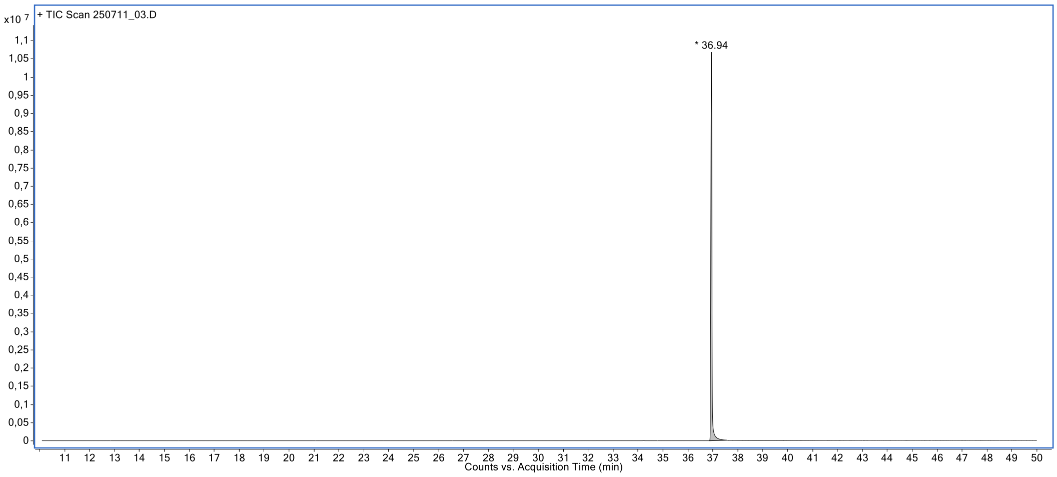


**Figure S3.** HS-SPME-GC-MS chromatogram of 3-sulfanylhexyl propionate. Method: 40 °C, 4 min; 5 °C/min → 210 °C, 0 min; 20 °C/min → 240 °C, 10.5 min; 1.2 mL/min helium (constant flow); column: 60 m × 0.25 mm ID, 1.0 µm $d_f$, Rxi-5Sil MS (Restek GmbH, Bad Homburg, Germany).

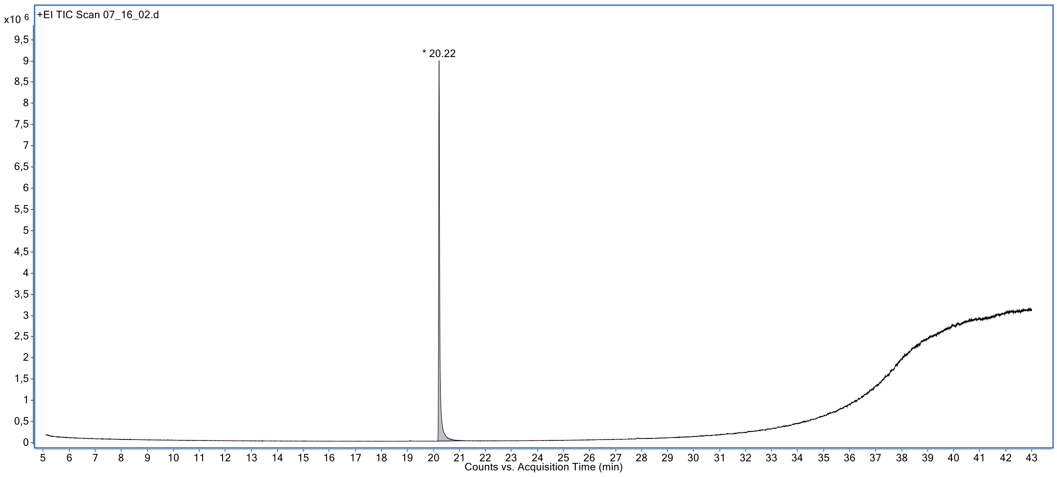


**Figure S43.** GC-MS chromatogram of 3-sulfanylhexyl propionate in DCM. Method: 40 °C, 3 min; 6 °C/min → 250 °C, 5 min; 1.2 mL/min helium (constant flow); column: 30 m × 0.25 mm ID × 0.25 µm $d_f$, Stabilwax-DA (Restek GmbH, Bad Homburg, Germany).

## 4. Mass spectra

### 4.1 3-Sulfanylhexyl butyrate

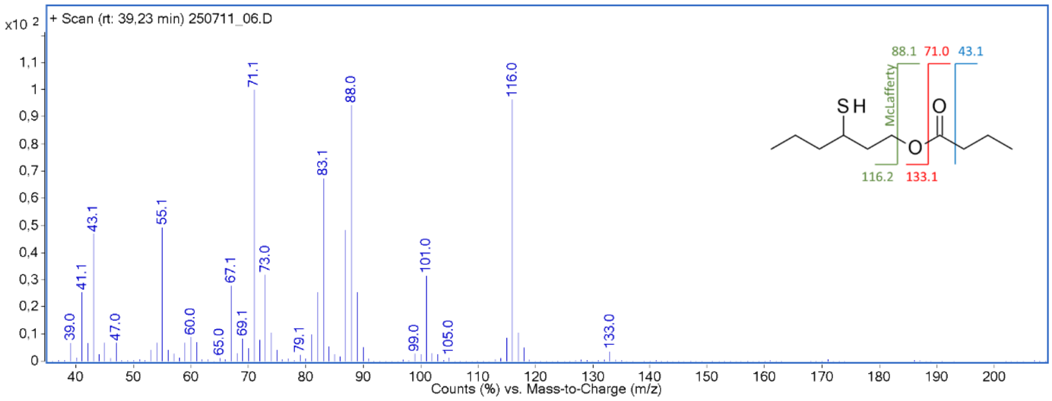


**Figure S5.** Mass spectrum (EI, 70 eV) of 3-sulfanylhexyl butyrate.

### 4.1 3-Sulfanylhexyl propionate

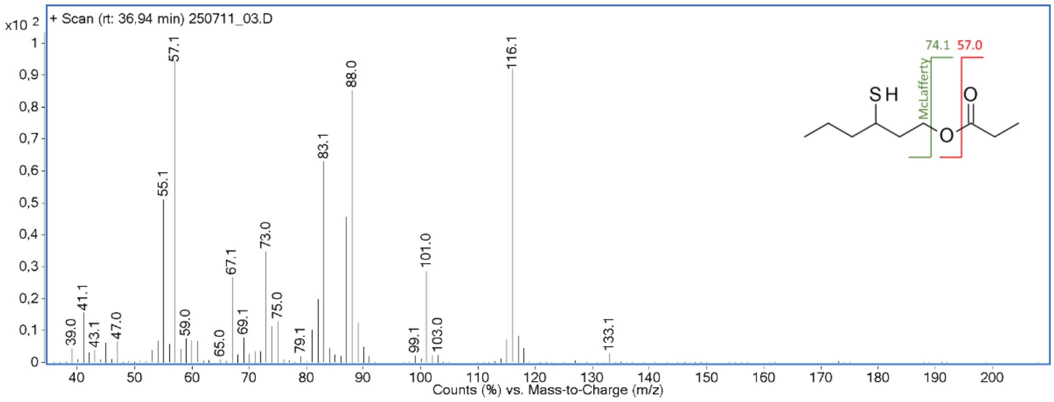


**Figure S6.** Mass spectrum (EI, 70 eV) of 3-sulfanylhexyl propionate.

## 5. NMR-spectra

### 5.1 3-Sulfanylhexyl butyrate

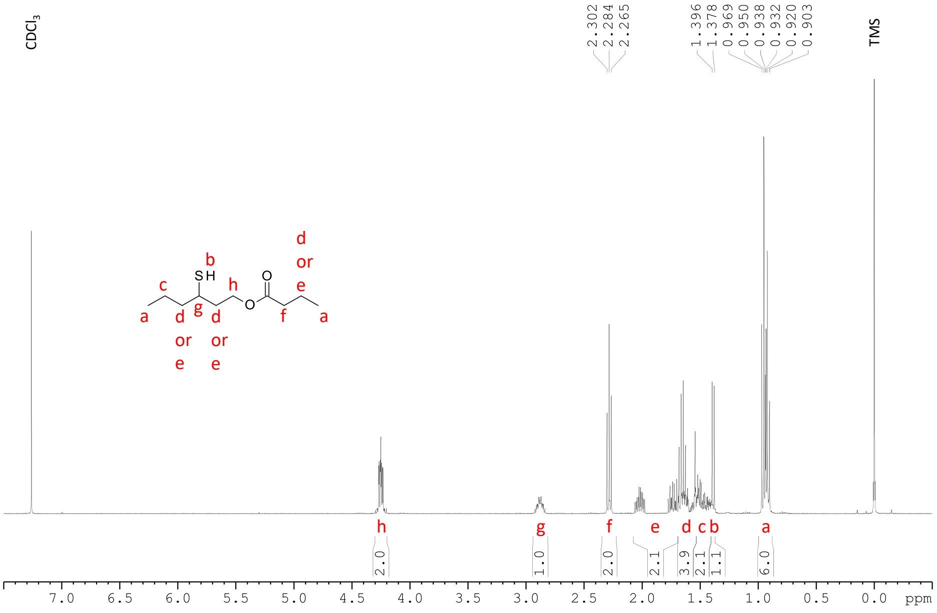

**Figure S7.** $^{1}H$ NMR (400 MHz, $CDCl_3$) of 3-sulfanylhexyl butyrate (3SHB).

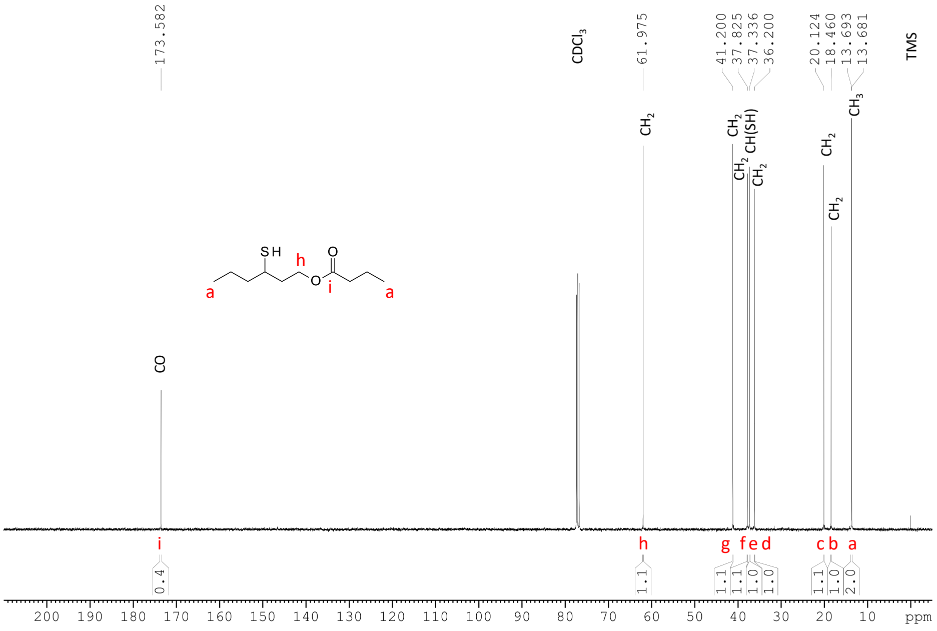

**Figure S8.** $^{13}C$ NMR (101 MHz, $CDCl_3$) of 3-sulfanylhexyl butyrate (3SHB).

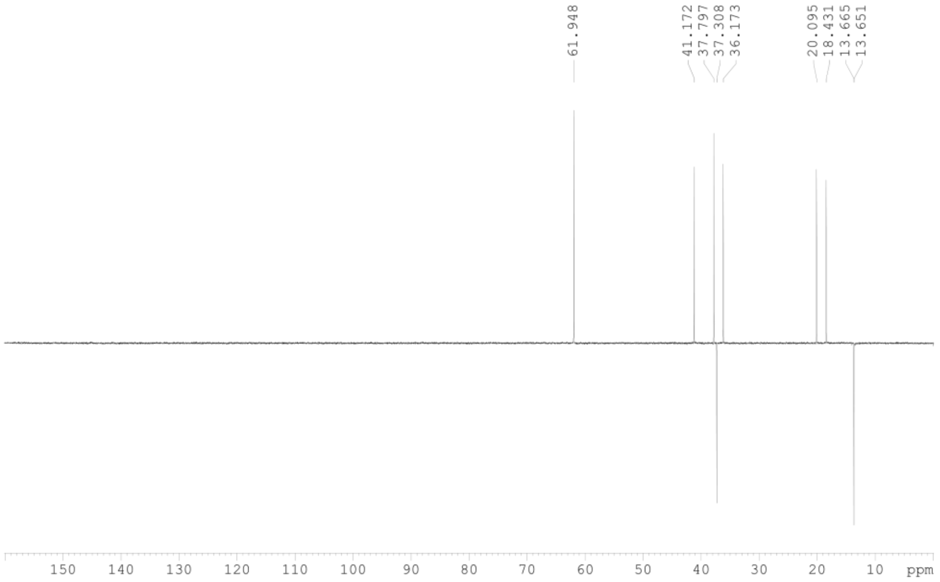


**Figure S9.** DEPT-135 spectrum (101 MHz, $CDCl_3$) of 3-sulfanylhexyl butyrate (3SHB).

## 5.2 3-Sulfanylhexyl propionate

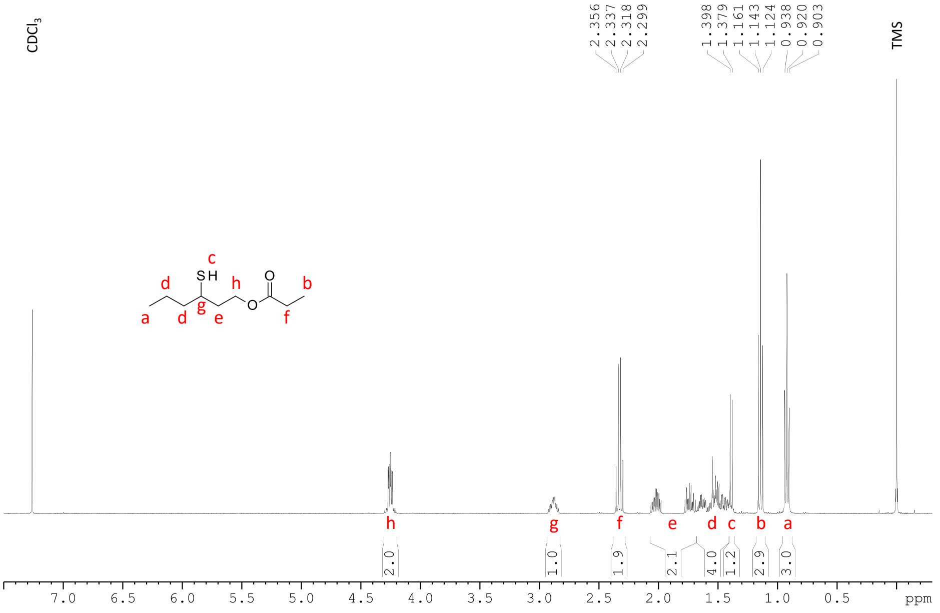


**Figure S10.** $^{1}$H NMR (400 MHz, $CDCl_3$) of 3-sulfanylhexyl propionate (3SHP).

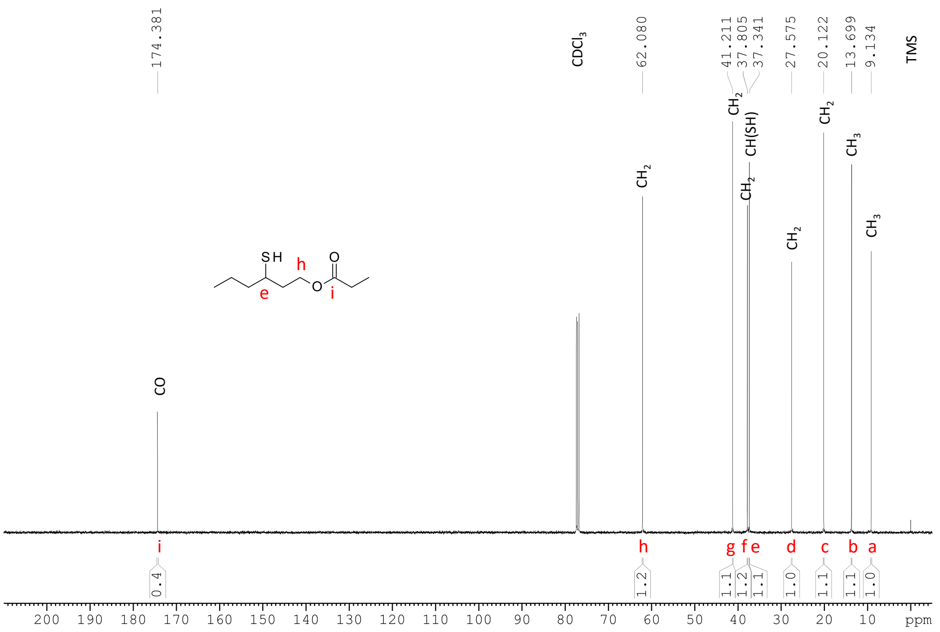


**Figure S11.** $^{13}$C NMR (101 MHz, CDCl$_3$) of 3-sulfanylhexyl propionate (3SHP).

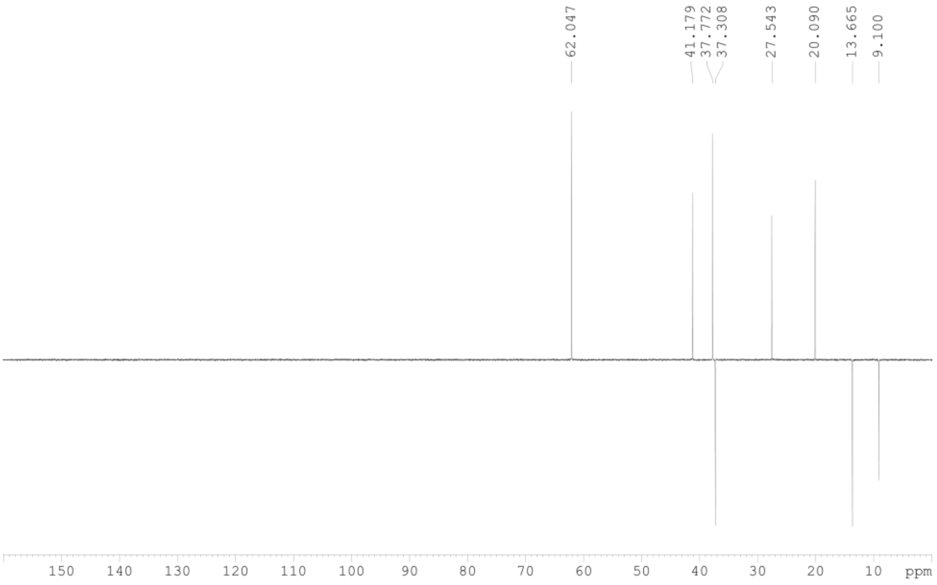


**Figure S12.** DEPT-135 spectrum (101 MHz, CDCl$_3$) of 3-sulfanylhexyl propionate (3SHP).

## 6. Sensory evaluation

**Table S2.** Concentration levels of thiols in ten water solutions prepared for the sensory analysis of odor threshold values.

| Thiols | Concentrations (ng·L$^{-1}$) of the thiols in water solutions | | | | | | | | | |
|---|---|---|---|---|---|---|---|---|---|---|
| | Glass 1 | Glass 2 | Glass 3 | Glass 4 | Glass 5 | Glass 6 | Glass 7 | Glass 8 | Glass 9 | Glass 10 |
| **3SH** | 0.0 | 0.0 | 0.0 | 16.0 | 32.0 | 32.0 | 62.8 | 124.4 | 124.4 | 374.5 |
| **3SHA** | 0.0 | 0.0 | 0.3 | 0.8 | 0.8 | 2.4 | 2.4 | 4.8 | 9.6 | 9.6 |
| **3SHP** | 0.0 | 0.0 | 76.7 | 76.7 | 76.7 | 149.7 | 149.7 | 299.4 | 449.0 | 748.4 |
| **3SHB** | 0.0 | 0.0 | 0.0 | 38.4 | 38.4 | 76.8 | 153.5 | 307.0 | 307.0 | 767.6 |

## 7. Fermentation trials with GMO

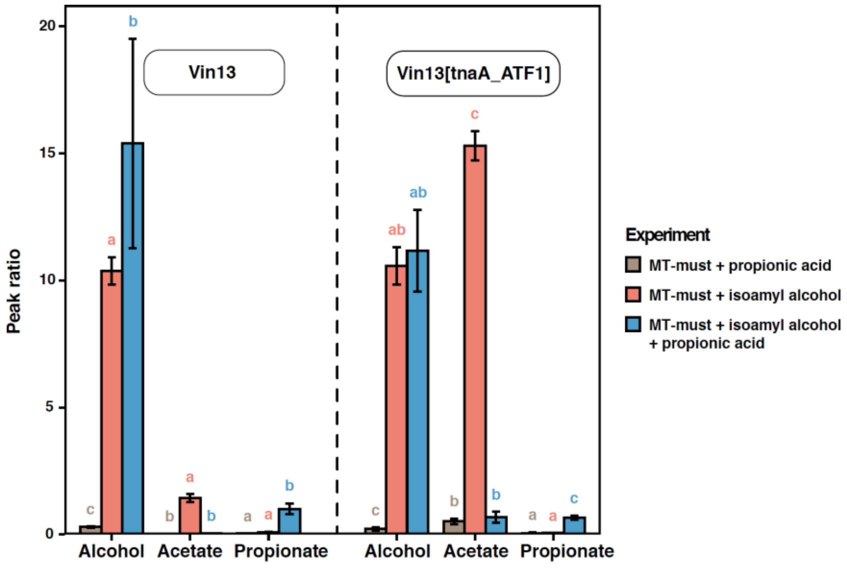


**Figure S13**. Resulting peak ratio of isoamyl alcohol, isoamyl acetate, and isoamyl propionate in fermentations with VIN13 and VIN13[tnaA_ATF1]. Superscript letters of the same compound with the same yeast indicate statistically significant differences ($p < 0.05$) as determined by one-way ANOVA followed by Tukey's post hoc test.

## 8. Preliminary fermentation trials with fatty acid addition

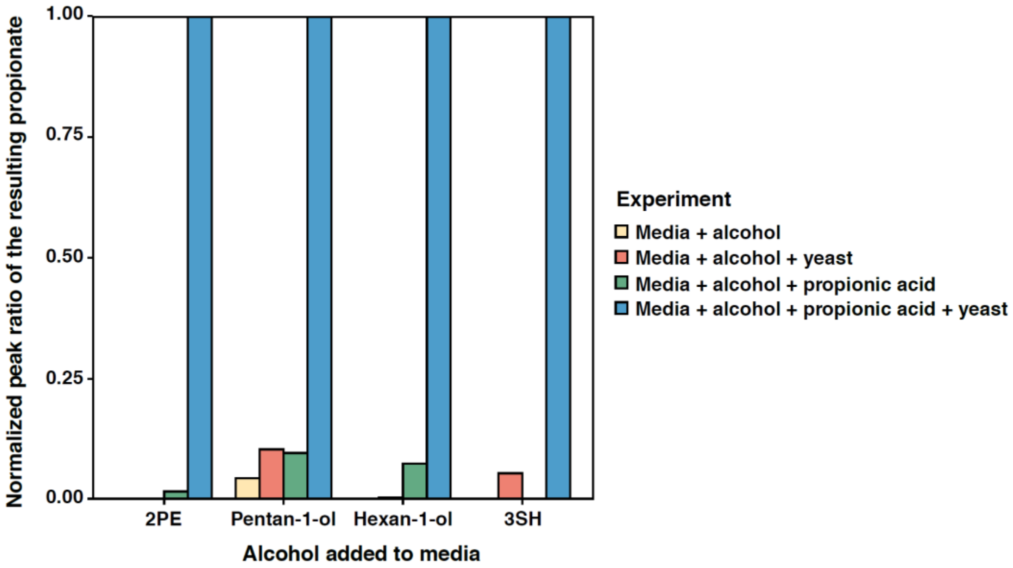


**Figure S14.** Normalized peak ratio of the formed propionates in fermentations with the addition of the alcohols 2-phenylethanol (2PE), pentan-1-ol, hexan-1-ol, or 3-sulfanylhexan-1-ol (3SH) to YPD media without fermentation (yellow), without fermentation but with the addition of propionic acid (green), and after fermentation with VIN13[tnaA_ATF1] without addition (red) and with the addition of propionic acid (blue).

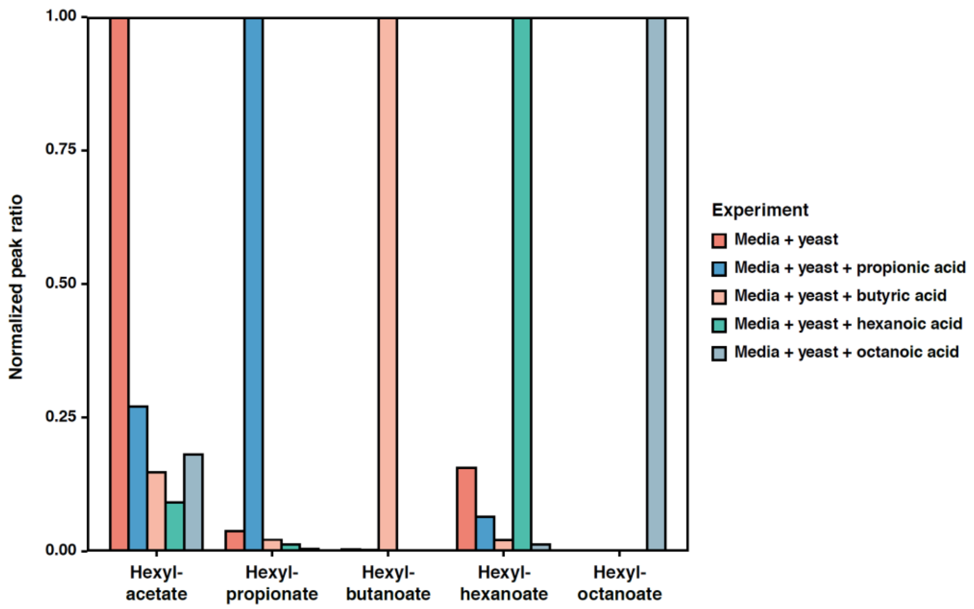


**Figure S15.** Normalized peak ratio of the formed esters in fermentation experiments (VIN13[tnaA_ATF1]) with hexan-1-ol and various fatty acids (propionic, butanoic, hexanoic, and octanoic) to investigate ester formation. Chemical esterification could be excluded by control samples (data not shown).

## 9. Spearman Correlation

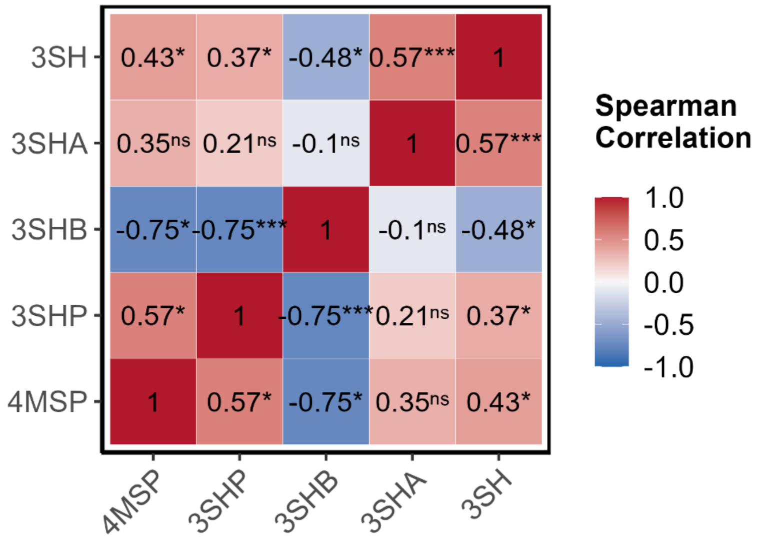


**Figure S16.** Spearman correlation matrix for thiol concentrations measured in Sauvignon blanc samples. Values indicate the correlation coefficient (r) along with significance levels: *p ≤ 0.05, **p ≤ 0.01, *** p ≤ 0.001, ns: not significant.

## 10. References

(1) van Wyk, N.; Kroukamp, H.; Espinosa, M. I.; von Wallbrunn, C.; Wendland, J.; Pretorius, I. S. Blending Wine Yeast Phenotypes with the Aid of CRISPR DNA Editing Technologies. *Int. J. Food Microbiol.* **2020**, 324, 108615.
(2) Badura, J.; van Wyk, N.; Zimmer, K.; Pretorius, I. S.; von Wallbrunn, C.; Wendland, J. PCR-based Gene Targeting in *Hanseniaspora uvarum*. *FEMS Yeast Res.* **2023**, 23, foad049.
(3) Badura, J.; van Wyk, N.; Brezina, S.; Pretorius, I. S.; Rauhut, D.; Wendland, J.; von Wallbrunn, C. Development of Genetic Modification Tools for *Hanseniaspora uvarum*. *Int. J. Mol. Sci.* **2021**, 22, 5437.
